# Functional Analysis of the Maize C-Repeat/DRE Motif-Binding Transcription Factor CBF3 Promoter in Response to Abiotic Stress

**DOI:** 10.3390/ijms160612131

**Published:** 2015-05-28

**Authors:** Jinliang Liu, Fengting Wang, Gang Yu, Xianghui Zhang, Chengguo Jia, Jianchun Qin, Hongyu Pan

**Affiliations:** College of Plant Sciences, Jilin University, Changchun 130062, China; E-Mails: jlliu@jlu.edu.cn (J.L.); wft1001@163.com (F.W.); chrisyu_gang@126.com (G.Y.); zhangxianghui@jlu.edu.cn (X.Z.); jiacg@jlu.edu.cn (C.J.)

**Keywords:** promoter, *ZmCBF3*, transgenic *Arabidopsis*, abiotic stress

## Abstract

The *ZmCBF3* gene is a member of AP2/ERF transcription factor family, which is a large family of plant-specific transcription factors that share a well-conserved DNA-binding domain. To understand the regulatory mechanism of *ZmCBF3* gene expression, we isolated and characterized the *ZmCBF3* promoter (P*ZmCBF3*). Three deletion fragments of P*ZmCBF3* were generated, C1–C3, from the translation start codon at position −1079, −638, and −234, and fused to the *GUS* reporter gene. Each deletion construct was analyzed by *Agrobacterium*-mediated stable transformation and expression in *Arabidopsis thaliana*. GUS expression assays indicated that the P*ZmCBF3* exhibited root-specific expression activity. A 234-bp fragment upstream of the *ZmCBF3* gene conferred a high level of GUS activity in *Arabidopsis*. Some *cis*-acting elements involved in the down-regulation of gene expression were detected in the promoter, encompassing positions −1079 to −234. P*ZmCBF3* was activated by cold stress. The MYCCONSENSUSAT elements (CANNTG) were responsible for the ability of P*ZmCBF3* to respond to cold stress. The results of the present study suggest that P*ZmCBF3* might play a role in cold tolerance in maize.

## 1. Introduction

Drought, high salinity, and low temperature are common stress conditions that have adverse effects on plant survival, growth, and reproduction. To continue growth and reproduction under such conditions of abiotic stresses, plants respond to unfavorable environments by using various developmental, physiological, and biochemical mechanisms. The C-repeat (CRT)/DRE (dehydration-responsive element) motif-binding transcription factors (CBFs) are identified as stress-responsive genes. Low temperatures can induce the expression of CBFs in plants, as well as enhance frost resistance [1,2]. The CBFs can specifically bind to the CRT/DRE element, which is located in the promoter region of cold-regulated genes and upregulates their expression and thus increases frost/freeze tolerance in plants [3].

The CBF gene family includes *CBF1*, *CBF2*, *CBF3*, and *CBF4*. These four genes play an important role in plants that improve resistance to cold, drought, and salt. Using the yeast one-hybrid system, AtCBF1, which specifically binds to CRT/DRE, was identified in an *Arabidopsis* gene library [1]. These sequences shared 88% similarity at the amino acid level. Five protein-binding DRE elements were identified in DREB1A (DRE-binding protein 1A), DREB1B, and DREB1C corresponding to CBF3, CBF1, and CBF2, respectively [4]. In 2002, the fourth member of the CBF family, CBF4, was identified [5]. In addition to *Arabidopsis*, *CBF* genes also exist in other plant species such as oilseed rape, barley, tomato, rice, rye grass, maize, and soybean [6,7,8,9,10,11,12]. Proteins belonging to the CBF family have a highly conserved DNA-binding domain called the AP2/ERF domain, which consists of ~60 amino acids. The domain is also found in CBF-like proteins of other plants [13]. The AP2/ERF domain plays an important role in recognizing and binding specifically to the CRT/DRE *cis*-acting elements to activate the corresponding gene expression [14].

Maize (*Zea mays* L.) is one of the world’s most important crops. In our previous study, the gene expression of maize leaves under drought and low-temperature stresses was analyzed by using gene chip technology (unpublished results), which showed the upregulation of *ZmCBF3*. Although the function of CBF proteins in plants under abiotic stresses has been previously reported, investigations on CBF promoters responding to the abiotic stress have not been performed. Studying the roles of promoters under different environmental stresses and plant hormone treatment will help us to better understand the regulation of CBF self-expression, and would be a significant guide for its actual production and transgenic breeding. In the present study, the upstream promoter sequence of the *ZmCBF3* gene was isolated and its function was analyzed by using 5′-end deletion and GUS activity assays.

## 2. Results

### 2.1. Promoter Sequence Analysis

**Figure 1:**
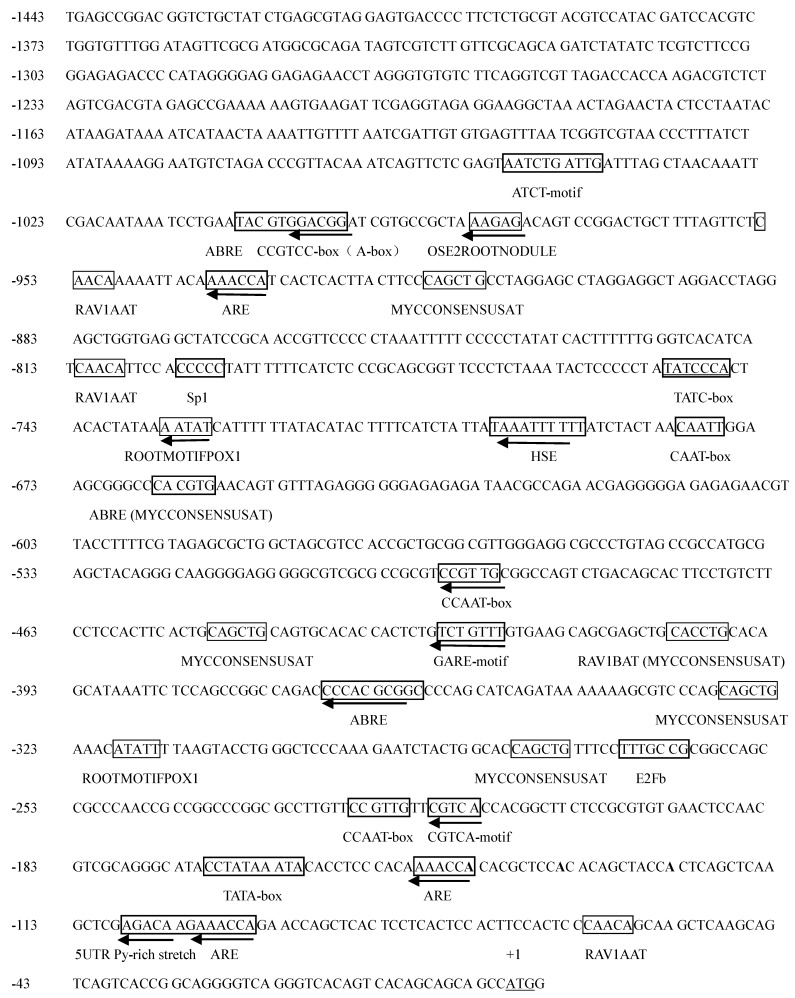
Nucleotide sequence and putative *cis*-acting elements of the *ZmCBF3* promoter (P*ZmCBF3*). The *cis*-acting elements are boxed, and arrows indicate their direction. The translation initiation codon is underlined.

To characterize the promoter of *ZmCBF3* (P*ZmCBF3*), the 5′-upstream sequence was cloned from maize (B73) genomic DNA. The cloned DNA region was 1443 bp in length (Figure 1) and was analyzed by using the PLACE and the PlantCARE databases. The putative *cis*-acting elements were identified and labeled (Figure 1), which were homologous to the *cis-*acting elements of other plants.

Three kinds of promoter elements known to be regulated by hormones in some plant genes were identified in the *PZmCBF3* sequence: three *cis-*acting elements involved in the abscisic acid responsiveness (ABREs); two *cis-*acting regulatory elements associated with MeJA-responsiveness (CGTCA-motif and TGACG-motif), and two *cis*-acting elements participating in gibberellin-responsiveness (TATC-box and GARE-motif).

Three putative regulatory motifs, which are involved in the activation of stress and defense genes in plants, were detected in the promoter region of the *ZmCBF3* gene. One was the *cis*-acting element involved in heat stress responsiveness (HSE), the other included two MYBHv1 binding sites (CCAAT-box), and the last one comprised seven MYCCONSENSUSAT elements (CANNTG).

Four kinds of putative root-specific elements were detected within the P*ZmCBF3*, which included four RAV1AAT (CAACA), two ROOTMOTIFPOX1 (CAACA), two RHERPATEXPA7 (KCACGW), and one OSE2ROOTNODULE (CTCTT) sites.

Other elements were also detected within the P*ZmCBF3* sequence, which included three *cis*-acting regulatory element (AREs) that were essential for anaerobic induction, one 5′-UTR Py-rich stretch (a *cis*-acting element conferring high transcription levels), one A-box (a *cis*-acting regulatory element), one CCGTCC-box (a *cis*-acting regulatory element related to meristem specific activation), one O_2_-site (*cis*-acting regulatory element involved in zein metabolism regulation), and several light responsive elements such as G-box, Sp1, GAG-motif, and ACE.

### 2.2. Basal Expression Analysis

**Figure 2:**
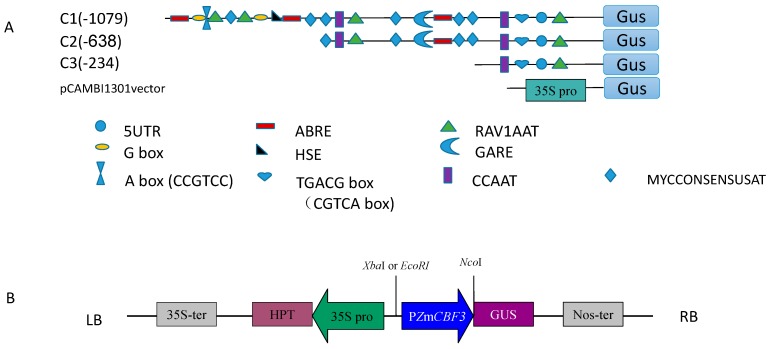
Transformation of *Arabidopsis* plants using P*ZmCBF3:GUS* constructs. (**A**) Schematic representation of the P*ZmCBF3* promoter constructs used to analyze GUS expression in *Arabidopsis* leaves. The serial 5′-deletion constructs of the P*ZmCBF3* promoter were fused to the GUS reporter gene in the vector, pCAMBIA1301; (**B**) Schematic representation of the P*ZmCBF3:GUS* construct. The insertion position of the *ZmCBF3* promoter in the vector is indicated with restriction enzyme sites (*Xba*I or *Eco*RI and *Nco*I). LB, left border; RB, right border; 35S-ter, CAMV 35S terminator; 35S pro, CAMV 35S promoter; GUS, β-glucuronidase gene; HPTII, hygromycinphosphotransferase (II) coding region; NOST, nopaline synthase terminator; P*ZmCBF3*, *ZmCBF3* promoter.

**Figure 3:**
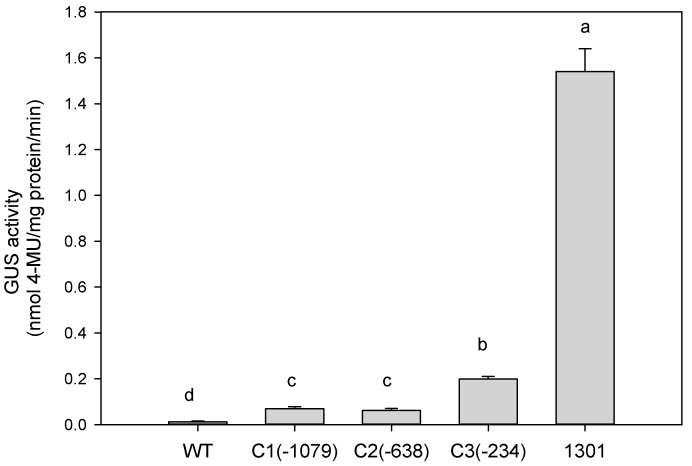
Basal expression analysis of GUS activity in transgenic *Arabidopsis* lines. GUS activity was analyzed fluorometrically and expressed as nmoles 4-methylumbelliferone (MU)/mg protein/min. Data are expressed as the mean ± standard deviation of three independent assays of *Arabidopsis* leaf extracts. Six-week-old *Arabidopsis* T3 plants and wild type (WT) were used for this analysis. Same letters indicate no significant differences at *p* ≤ 0.05 by Duncan test. C1(−1,079), C2(−638), and C3(−234) represent different deletion promoters. Different letters (a–d) mean statistical differences between treatments.

**Figure 4:**
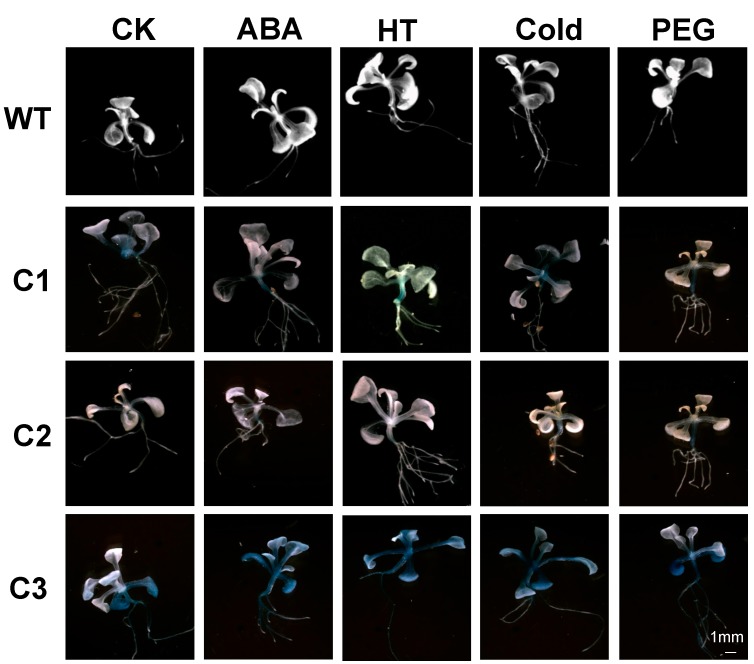
Histochemical expression of the β-glucuronidase (GUS) reporter gene in transgenic *Arabidopsis* and wild type (WT) in response to abiotic stresses and abscisic acid (ABA). Two-week-old *Arabidopsis* T3 plants were subjected to ABA, high temperature (HT), cold (4 °C), and drought (20% PEG6000) treatments for 6 h. Control (CK) plants were treated with water. C1, C2, and C3 represent different deletion promoters. Scale bar for all treatments are shown in C3-PEG. Scale bars: 1 mm.

To determine the minimal necessary region of P*ZmCBF3*, three promoter deletion fragments were fused with the GUS reporter gene in pCAMBIA1301 for agro-infiltration into *Arabidopsis* (Figure 2). The expression of each P*ZmCBF3*:*GUS* fusion gene was examined in the leaves of 15 independent T3 transgenic *Arabidopsis* plants. As shown in Figure 3, deletion constructs with C3 showed much higher GUS activity than that of the C1 and C2 promoters in the leaves of transgenic *Arabidopsis* plants (Figure 3 and Figure 4). C1 and C2 promoter-mediated GUS activity showed no significant difference. Compared to GUS activity driven by cauliflower mosaic virus (CaMV) 35S promoter (as positive control), P*ZmCBF3* showed lower activity in leaves. In addition, C3-mediated GUS activity was accounting for only 13% of the CaMV 35S promoter-mediated GUS activity. These results indicated that the leaves of the T3 transgenic *Arabidopsis* plants had very low P*ZmCBF3* activity.

### 2.3. Abiotic Stress-Induced Expression Analysis

To verify the role of stress-responsive *cis*-acting elements in P*ZmCBF3*, deletion promoter constructs were fused to the GUS reporter gene (Figure 2). GUS activity of the different deletion promoters in transgenic *Arabidopsis* leaves after treatment with high temperature (HT), cold, ABA, and PEG6000 were then examined.

**Figure 5:**
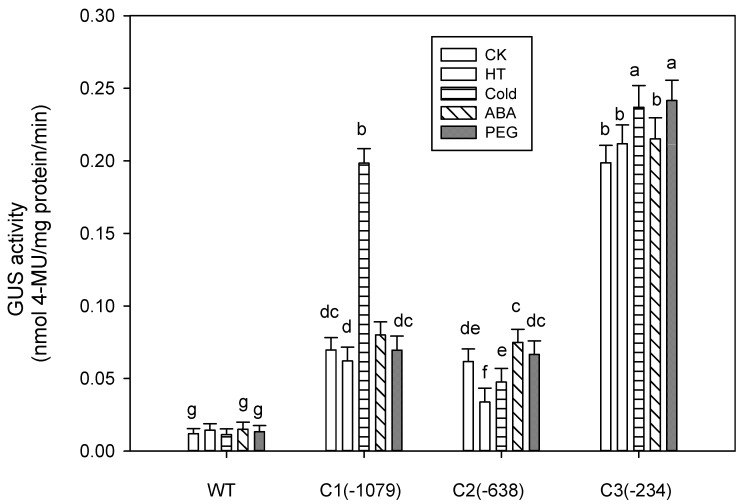
Abiotic stress-induced GUS activity analysis of transgenic *Arabidopsis* lines. Six-week-old *Arabidopsis* T3 plants and wild type (WT) were subjected to ABA, high temperature (HT), cold (4 °C), and drought (20% PEG6000) treatments for 6 h. Control (CK) plants were treated with water. GUS activity was analyzed fluorometrically and expressed as nmoles 4-methylumbelliferone (MU)/mg protein/min. Data are expressed as the mean ± standard deviation of three independent assays involving *Arabidopsis* leaf extracts. The same letters indicate no significant differences at *p* ≤ 0.05 by Duncan’s test. Different letters (a–g) mean statistical differences between treatments.

Compared to the control leaves (untreated plants), GUS activity in C2-transformed leaves decreased by 0.547-fold after HT treatment (Figure 5). However, marginal induction of GUS activity was observed in C1- and C3-transformed *Arabidopsis* leaves. Under cold stress, C1- and C3-mediated GUS activity increased by 2.8- and 1.19-fold, respectively, whereas GUS activity of C2-transformed plants did not significantly change (Figure 5). As shown in Figure 5, GUS activity of deletion promoters slightly increased after ABA and PEG treatment. GUS activity of the C1 and C2 promoters increased by 1.15- and 1.21-fold respectively, after ABA treatment. C3-mediated GUS activity significantly increased (by about 1.22-fold) with PEG treatment.

### 2.4. Tissue-Specific Expression Analysis

**Figure 6:**
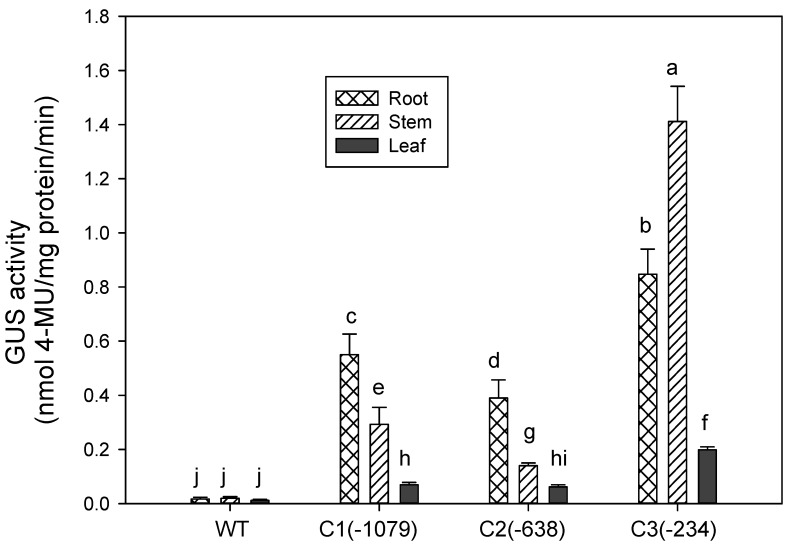
Tissue-specific expression analysis of GUS activity among different transgenic *Arabidopsis* lines. GUS activity was examined in six-week-old *Arabidopsis* T3 plants and wild type (WT). GUS activity was analyzed fluorometrically and expressed as nmoles 4-methylumbelliferone (MU)/mg protein/min. Data are expressed as the mean ± standard deviation of three independent assays involving *Arabidopsis* leaf extracts. The same letters indicate no significant differences at *p* ≤ 0.05 by Duncan’s test. Different letters (a–j) mean statistical differences between treatments.

In our early experiments, histochemical staining was employed to determine GUS activity of the deletion promoters in transgenic *Arabidopsis* (Figure 4). In Figure 4, GUS staining was mainly detected in the stems and roots. The promoter apparently shows tissue-specific expression. To test this hypothesis, GUS activity of P*ZmCBF3* was determined in the transgenic *Arabidopsis* roots, stems, and leaves by fluorometric analyses. GUS activity of the C1 and C2 promoters was highest in the roots, followed by the stems (Figure 6). C3-mediated GUS activity was highest in the stems, which was 1.67-fold that of the root (Figure 6). These results were consistent with that of GUS staining.

## 3. Discussion

### 3.1. Basal Expression Analysis of the Deletion Promoters

To determine the function of *cis*-acting elements, deletion fragment analysis of the P*ZmCBF3* was performed. The deletion promoter constructs were then transformed into *Arabidopsis* by using *Agrobacterium*-mediated transformation. Several studies have earlier shown that promoter activity gradually decreases with shorter promoter lengths [15,16]. However, in the present study, the smallest promoter fragment C3(−234) showed the highest activity among the deletion promoters (Figure 3). This finding suggests that the sequence between −1079 and −234 bp of the P*ZmCBF3* might contain *cis*-acting elements that are involved in the downregulation of gene expression. This phenomenon has also been observed in other promoters [17]. Dalal *et al.* reported that the strength of the tissue-specific promoter ShCYC-B drastically decreased in deletion mutants, and further deletion of the promoter sequence at 5′ end resulted in the highest activity [18].

### 3.2. Abiotic Stress-Induced Expression Analysis

The deletion promoters of *ZmCBF3* exhibited inducible expression in response to different stress stimuli (HT, cold, ABA, and PEG; Figure 5). Heat shock elements (HSEs) are the binding sites for the trans-active heat shock factor (HSF), and three or more units are required for efficient binding [19,20]. In the present study, sequence analysis of P*ZmCBF3* showed that there was one HSE within the segment encompassing −691 to −701 bp. GUS activity in C1-transformed *Arabidopsis* leaves was almost not induced by high temperature (Figure 5). The results suggest that HSE did not affect the C1 promoter in response to high temperature. However, negative regulatory elements might be located in the promoter region, covering −638 to −1079 bp, which induced a decrease in GUS activity of C2-mediated after HT treatment.

After cold treatment, GUS activity of C1-mediated plants increased by 2.8-fold (Figure 5), which may be attributed to MYCCONSENSUSAT elements (CANNTG). Sequence analysis identified seven MYCCONSENSUSAT elements within P*ZmCBF3*. The MYC recognition sequence was the binding site of ICE1, which encodes a MYC-like bHLH transcriptional activator that specifically binds to multiple MYC DNA regulatory elements in the CBF3 promoter, stimulating CBF3 transcription [21]. However, GUS activity of C2-transformed plants did not significantly change, which suggests that part of the seven elements played an important role in regulating gene expression during cold stress. The MYCCONSENSUSAT elements present between −638 to −234 bp might not play an important role for the promoter.

ABRE (PyACGTG/TC) is a well-studied *cis*-element involved in ABA-induced gene expression [22]. Promoters sequence analysis has revealed that ABA-responsive gene expression requires multiple ABA-responsive elements (ABREs or CE3) [23,24]. The ABRE-binding protein/factor (AREB/ABF) family is composed of nine members in *Arabidopsis* [25,26]. Sequence analysis of P*ZmCBF3* identified three ABREs within the promoter. We hypothesized that ABREs play an important role in increasing GUS activity under ABA treatment. ABA is one of the most important signaling molecules; furthermore, it is also a cross-talking signal molecule. In plants, osmotic stress-responsive transcriptional regulation depends mainly on two pathways: ABA-dependent and ABA-independent pathways [27]. In the present study, GUS activity of P*ZmCBF3* showed the same changes under ABA and PEG stress, suggesting that the expression of *ZmCBF3* might be an ABA-dependent pathway under PEG stress.

### 3.3. Expression of PZmCBF3 Is Tissue-Specific

Most AP2/ERF transcription factors exhibit root-specific expression [28]. Research investigations on root-specific promoters and root-specific *cis-*acting elements are limited. For example, tissue-specific expression of *TobRB7* in tobacco involves the root meristem and central cylinder regions and encodes a cytosol protein [29]. Other root-specific promoters have also been identified such as AtEXPA7, AtEXPA18 [30], EXPANSIN A [31,32], and PAL [33]. In addition, some root-specific *cis*-acting elements have been detected in root-specific promoters. The Atlg73160 promoter sequence [29] consists of root-specific regulatory elements such as ASF1MOTIFCAMV, ARFAT, OSE1, OSE2, SURE core, and RAV1AAT. RHERPATEXPA7 and ROOTMOTIFPOX1 have also been identified in other promoters [34,35].

The present study has shown that P*ZmCBF3* is highly expressed in the root (Figure 4 and Figure 6). Sequence analysis has identified four RAV1AAT (−954 to −949 bp, −812 to −807 bp, −403 to −397 bp, −62 to −57 bp), two ROOTMOTIFPOX1 (−734 to −729 bp, −319 to −314 bp), two RHERPATEXPA7 (−994 to −988 bp, −664 to −658 bp), and one OSE2ROOTNODULE (−973 to −968 bp) in the *ZmCBF3* promoter. Two RAV1AAT (−403 to −397 bp, −62 to −57 bp) and one ROOTMOTIFPOX1 (−319 to −314 bp) were present in the C2 promoter, and only one RAV1AAT (−62 to −57 bp) was detected within the C3 promoter. Figure 6 shows that the highest C1- and C2-mediated GUS activity was observed in the roots of transgenic *Arabidopsis*. However, GUS activity of C3-mediated expression was lower than that of other tissues and organs, in which most of the root-specific regulatory elements of the promoter were deleted. This finding suggests that RAV1AAT and ROOTMOTIFPOX1 play an important role in the root-specific expression of P*ZmCBF3*.

## 4. Experimental Section

### 4.1. Plant Materials, Growth Condition, and Bacterial Strains

Maize plants (cultivar B73) were raised in pots containing soil mix (soil:vermiculite, 3/1, *v*/*v*) under greenhouse conditions (16/8 h photoperiod; 26 °C), with three plants per pot. The fifth expanding leaves were sampled for genomic DNA extraction. *Arabidopsis thaliana* (L.) ecotype Col-0 plants were grown in a soil mix (2:1:1 mixture of vermiculite, peat moss, and perlite) and placed at 22/19 ± 2 °C (light/dark, 16/8 h photoperiod) with a light intensity of approximately 100–120 µmol·m^−2^·s^−1^ in a growth chamber. *Escherichia coli* strain DH5α was used to clone and propagate all recombinant plasmid vectors. *Agrobacterium tumefaciens* strain EHA105 was used for infiltrating *A. thaliana* leaves.

### 4.2. Genomic DNA Extraction

Total DNA was isolated from young leaves using CTAB (cetyltrimethylammonium bromide) as previously described [36], with slight modifications. Approximately 0.1 g of leaf powder was added to 0.6 mL of pre-warmed (65 °C) extraction buffer (3% (*w*/*v*) CTAB, 2% (*w*/*v*) polyvinylpyrrolidone, 1 M Tris–HCl (pH 8.0), 20 mM EDTA, 1.4 M NaCl, and 2% (*v*/*v*) β-mercaptoethanol) and incubated for 30 min at 65 °C with occasional mixing. The slurry was mixed by inversion with 0.6 mL of chloroform/isoamyl alcohol (24/1, *v*/*v*) and centrifuged for 10 min at 9600× *g*. The upper phase was recovered and two volumes of isopropanol was added and incubated for 2 h at −20 °C. The suspension was centrifuged for 10 min at 9600× *g*, and the pellet was dried and dissolved in 50 mL of deionized H_2_O. The integrity of genomic DNA was assessed on 1% (*w*/*v*) agarose gel by electrophoresis followed by visualization with ethidium bromide staining.

### 4.3. Isolation of the ZmCBF3 Gene Promoter

Using gene chip technology, we obtained the *ZmCBF3* gene. The upstream sequence of the gene was obtained after aligned and analyzed at the MaizeGDB website (http://maizegdb.org/). The specific primers were designed according to the 5′ flanking sequence of *ZmCBF3*. The *ZmCBF3* promoter (P*ZmCBF3*) was amplified from maize genomic DNA by PCR in a mixture of 1 µL of each primer (10 µM, Pcbf-F and Pcbf-R, Table 1), 1.0 µL 5-mM dNTP, 2.5 µL 10× buffer, 0.5 µL of Taq DNA polymerase (Takara, Otsu, Japan), 0.5 µL DNA, and 18.5 µL sterile H_2_O. The PCR conditions were as follows: initial denaturation at 94 °C for 4 min; followed by 30 cycles of denaturation at 94 °C for 40 s, annealing at 54 °C for 40 s, and extension at 72 °C for 1 min 30 s; and a final extension at 72 °C for 10 min. PCR products were analyzed on 1% agarose/ethidium bromide gel, purified using TIANquick Midi Purification Kit (DP209, Beijing, China) [37], cloned into pMD18-T cloning vector, and then transformed into *E. coli* strain DH5a. The positive clones were sequenced using an automated DNA sequencer at the Beijing Genomics Institute (Beijing, China).

**Table 1:** Primers used for PCR.

Oligo Name	Sequence (5′→3′)
Pcbf-F	TGAGCCGGACGGTCTGCTAT
C2-638	GGAATTCAGAGATAACGCCAGAACGAG
C3-234	GGAATTCGCCTTGTTCCGTTGTTCG
Pcbf-R	TGCCATGGCTGCTGCTGTGACTGTGA
Hyg-F	GATGTTGGCGACCTCGTATT
Hyg-R	TCGTTATGTTTATCGGCACTTT

### 4.4. Sequence Analysis

Putative *cis*-acting elements and their positions in the P*ZmCBF3* promoter were identified using PLACE [38] and PlantCARE [39].

### 4.5. Construction of the PZmCBF3 Deletion Constructs

To amplify the 5ʹ upstream sequence of the P*ZmCBF3* gene more easily, the upstream primer did not contain a restriction site, while the downstream primer contained a *Nco*I restriction site. The P*ZmCBF3* region from −1079 to +1, designated C1 (−1079), was obtained by restriction enzyme (*Xba*I and *Nco*I) digestion of the clone pMD18-T:P*ZmCBF3*. Two deletions, P2 (−638 bp) and P3 (−234 bp) (Figure 2A), were generated by PCR amplification, as described above, using the clone pMD18-T:P*ZmCBF3* as template. Forward primers containing an *Eco*RI restriction site, and a common reverse primer containing a *Nco*I restriction site (Table 1). The binary vector pCAMBIA1301 was used to make all plasmid constructs (Figure 2B).

The CaMV 35S promoter in front of the β-glucuronidase (GUS) gene was excised through digestion and replaced with one of the promoter fragments. Promoter fragments inserted into all plasmid constructs was confirmed by sequencing, and the correct plasmid constructs of P*ZmCBF3:GUS* were transformed into *Agrobacterium* strain EHA105 by the freeze-thaw method [40].

### 4.6. Arabidopsis Plant Transformation with Different PZmCBF3:GUS Constructs

Promoter activity can be studied via instantaneous expression, which provides rapid results, but quantitative comparisons of promoter activity are error-prone because the number of infected cells is difficult to control. So in this study, we chose to study the promoter activity by transforming *Arabidopsis* plants to get stable expression. The pCAMBIA1301 vectors containing the promoter-GUS fusion construct was transferred into *Agrobacterium* strain EHA105 for transformation into *Arabidopsis* (Figure 2B) as described previously [41]. pCAMBIA1301 was the positive control. *Arabidopsis* blooms were inoculated by vacuum infiltration with *Agrobacterium* in Murashige and Skoog medium (MS) medium with 5% sucrose and 0.05% Silwet L-77. The transgenic *Arabidopsis* T0 was grown on MS agar plates supplemented with 25 mg/L hygromycin B and then the screened transgenic plants were screened again by PCR with primers from the hygromycin-coding region (Hyg-F and Hyg-R, Table 1). The transgenic *Arabidopsis* T1 and T2 were also screened on MS agar plates supplemented with 25 mg/L hygromycin B. At the same time, the transgenic strains, from which the ratio of resistant plants and sensitive plants was 3:1, were analyzed.

### 4.7. Abiotic Stress Treatments

For each treatment and control, 100 transgenic Arabidopsis T3 plants were grown on MS agar plates supplemented with 25 mg/L hygromycin B at 22 °C for 2 and 6 weeks then used for stress treatment experiments. Two-week-old transgenic Arabidopsis T3 and wild type (WT) were used for histochemical analysis and 6-week-old plants were used for the fluorescence measuring.

To characterize the induction of promoter activity by the stress-responsive and ABA, the plants were removed from MS agar plates and their roots soaked in water, solutions of abscisic acid (ABA, 100 µM) or polyethylene glycol solution (PEG6000, 20%). High temperature (HT) and cold treatment was conducted under dim light by exposing plant roots soaked in water to a temperature of 37 and 4 °C. For the dehydration experiments, the plant roots were soaked in Polyethylene glycol solution (PEG6000, 20%) to simulate drought. The transgenic plants were soaked in water as control. In each case, plants were subjected to treatment for 6 h then frozen in liquid nitrogen.

### 4.8. Histochemical and Fluorometric Analyses of GUS Activity

Histochemical analysis of GUS activity was performed on 2-week-old transgenic *Arabidopsis* T3 stress-treated and control plants. The frozen plant material was incubated with 1 mM 5-bromo-4-chloro-3-indolyl-b-d-glucuronide (X-Gluc) in 50 mM phosphate buffer (pH 7.0). After staining, chlorophyll was cleared from tissues by incubating in 70% ethanol [42]. The 6-week-old transgenic *Arabidopsis* T3 were used to determine GUS enzyme activity by measuring the fluorescence of 4-methylumbelliferone produced by GUS cleavage of 4-methylumbelliferyl-b-d-glucuronide (4-MUG) [42]. GUS activity is expressed as nanomoles of methylumbelliferone per minute per milligram of protein. Protein amount was determined using a Protein Assay kit (Bio-Rad, Hercules, CA, USA) using bovine serum albumin as a standard.

### 4.9. Data Analysis

All measurements were repeated at least three times, and the results were expressed as mean values. Error bars shown in figures represent standard deviation (SD). The means of three replicates of three independent samples were analyzed by Duncan test at the 5% probability level.

## 5. Conclusions

GUS expression assays indicated that the *ZmCBF3* gene exhibited root-specific expression. A 234-bp fragment upstream of the *ZmCBF3* gene coding sequence confers a high level of GUS expression in *Arabidopsis*. Some *cis*-acting elements involved in down-regulation of gene expression might exist in the sequence of the promoter from −1079 to −234 bp. The MYCCONSENSUSAT elements (CANNTG) were responsible for the ability of P*ZmCBF3* to be induced by cold stress. P*ZmCBF3*-mediated GUS expression was an ABA-dependent pathway under PEG stress conditions, in which ABREs played an important role.
